# Long‐Term Change in Bone Mineral Density in Women Living With HIV: A 10‐Year Prospective Controlled Cohort Study

**DOI:** 10.1002/jbm4.10761

**Published:** 2023-07-05

**Authors:** Heather M. Macdonald, Evelyn J. Maan, Claudie Berger, Hélène C. F. Côte, Melanie C. M. Murray, Neora Pick, Jerilynn C. Prior, for the CIHR Team in Cellular Aging and HIV Comorbidities in Women and Children: CARMA

**Affiliations:** ^1^ Active Aging Research Team University of British Columbia Vancouver BC Canada; ^2^ Department of Family Practice Faculty of Medicine, University of British Columbia Vancouver BC Canada; ^3^ Oak Tree Clinic, BC Women's Hospital and Health Centre Vancouver BC Canada; ^4^ Research Institute of the McGill University Health Centre Montreal QC Canada; ^5^ Department of Pathology & Laboratory Medicine University of British Columbia Vancouver BC Canada; ^6^ Centre for Blood Research Faculty of Medicine, University of British Columbia Vancouver BC Canada; ^7^ Women's Health Research Institute Vancouver BC Canada; ^8^ Department of Medicine, Division of Infectious Diseases University of British Columbia Vancouver BC Canada; ^9^ Centre for Menstrual Cycle and Ovulation Research, Department of Medicine, Division of Endocrinology University of British Columbia Vancouver BC Canada; ^10^ School of Population and Public Health Faculty of Medicine, University of British Columbia Vancouver BC Canada

**Keywords:** HIV, BONE MINERAL DENSITY CHANGE, WOMEN, ANTIRETROVIRAL THERAPY, CAMOS

## Abstract

Women living with HIV (WLWH) may be at higher risk for osteoporosis and fragility fractures. However, limited prospective data describe long‐term trajectories of bone mineral density (BMD) in WLWH versus women without HIV. Thus, in this prospective study, we aimed to compare 10‐year change in areal BMD (aBMD) between WLWH (*n* = 49; 36.8 ± 8.8 years; 96% pre/perimenopausal) and HIV‐negative women (population‐based controls; *n* = 49; 41.9 ± 9.2 years; 80% pre/perimenopausal). In an exploratory analysis, we compared fracture history between WLWH and controls. Outcomes were lumbar spine (L_1_ to L_4_), total hip, and femoral neck aBMD at baseline and follow‐up, which occurred at 13 and 10 years in WLWH and controls, respectively. We fit multivariable regression models to compare baseline and 10‐year change in aBMD between groups, adjusting for osteoporosis risk factors. Within WLWH, we examined associations between aBMD and HIV‐related factors, including combination antiretroviral therapy (cART) duration. WLWH were diagnosed 6.5 ± 3.7 years before baseline, 80% were on cART for 241 ± 142 weeks, and 49% had HIV plasma viral load <40 copies/mL. Before and after adjusting for osteoporosis risk factors, baseline and 10‐year change in aBMD did not differ between WLWH and controls at any site. At baseline, more WLWH than controls reported a history of low‐trauma fracture (30% versus 10%, *p* < 0.05) and major osteoporotic fracture (17% versus 4%, *p* < 0.05). During follow‐up, the number of WLWH and controls with incident fragility fracture was not significantly different. Lifetime cART duration and tenofovir use were not associated with aBMD 10‐year percent change. Higher CD4 count at baseline was positively associated with femoral neck aBMD 10‐year percent change. Long‐term aBMD change in this small WLWH cohort paralleled normal aging, with no evidence of influence from cART use; however, these results should be interpreted with caution given the small sample size. Larger cohort studies are needed to confirm these findings. © 2023 The Authors. *JBMR Plus* published by Wiley Periodicals LLC on behalf of American Society for Bone and Mineral Research.

## Introduction

As life expectancy in persons living with HIV (PLWH) nears that of HIV‐negative adults,^(^ ^1^ ^)^ there is greater focus on prevention and treatment of aging comorbidities in this population,^(^ ^1^, ^2^ ^)^ including osteoporosis^(^ ^3^, ^4^, ^5^ ^)^ and fragility fractures.^(^ ^6^, ^7^, ^8^ ^)^ Lower bone mineral density (BMD)^(^ ^3^, ^8^ ^)^ and higher fracture prevalence^(^ ^7^, ^8^, ^9^ ^)^ have been described in PLWH as compared with persons living without HIV. However, most investigations of BMD in PLWH have been either cross‐sectional comparisons between PLWH and HIV‐negative adults, or short‐term longitudinal designs with a focus on changes in BMD in PLWH after initiation of combination antiretroviral therapy (cART).^(^ ^10^, ^11^ ^)^ In a meta‐analysis of cross‐sectional studies, areal BMD (aBMD) as measured with dual‐energy X‐ray absorptiometry (DXA) was 3% to 5% lower in PLWH compared with controls, which was largely explained by lower body weight in PLWH.^(^ ^10^ ^)^ Similarly, we previously reported similar aBMD between women living with HIV (WLWH) and population‐based controls without HIV; however, WLWH reported more lifetime fragility fractures.^(^ ^12^ ^)^ In short‐term longitudinal studies, declines in aBMD are typically greatest in the first 48 to 96 weeks after cART initiation and stabilize thereafter.^(^ ^13^, ^14^, ^15^, ^16^ ^)^

What is less clear is the trajectory of change in aBMD over time frames of 10 years and longer, particularly in women. Erlandson and colleagues conducted the longest study to date of aBMD change among women and men living with HIV; participants underwent DXA scans every 6 to 12 months for up to 10 years (median follow‐up 4.6 years).^(^ ^17^ ^)^ Although significant sex differences were observed in the trajectory of aBMD change, the lack of an HIV‐negative comparison group meant it was not possible to determine whether these trajectories differed from or mirrored those of normal aging.

Therefore, our primary aim in this prospective study was to compare aBMD change across 10 years in WLWH, as compared with healthy age‐matched women from the population‐based Canadian Multicentre Osteoporosis Study (CaM*os*) in the same region. Our secondary aims were to explore associations between aBMD change and HIV‐related clinical characteristics among WLWH, and differences in fracture incidence between WLWH and HIV‐negative controls.

## Materials and Methods

### Study design and participants

This prospective study includes data from CARMA‐OSTEO, the bone health substudy of the CARMA (Children and Women: AntiRetrovirals and Markers of Aging) study, a prospective observational investigation of the effects of HIV and antiretroviral medications on cellular aging among children and women living with or exposed to HIV compared with HIV‐negative controls.^(^ ^18^, ^19^ ^)^ WLWH in CARMA‐OSTEO were originally participants in a cross‐sectional multicenter Canadian study of aBMD and prevalent fragility fracture, with data collected between May 2001 and September 2003^(^ ^12^ ^)^ (baseline for the present analysis). Cisgender WLWH who were still accessing care at the Oak Tree Clinic, a specialized pediatric and adult interdisciplinary HIV clinic located at the BC Women's Hospital + Health Centre in Vancouver, were then invited to participate in the CARMA‐OSTEO study, between July 2013 and August 2017 (follow‐up for the present analysis).^(^ ^20^ ^)^ The British Columbia (BC) Children's and Women's Research Ethics Board (H08–02018 and H09–02867) approved all procedures.

HIV‐negative cisgender women (controls) were selected for this analysis from the Vancouver CaM*os* cohort. CaM*os* is a population‐based longitudinal study of osteoporosis and fragility fractures initiated in 1995.^(^ ^21^ ^)^ Participants included 9423 community‐dwelling men and women who were at least 25 years old and lived within a 50‐km radius of one of nine Canadian cities, including Vancouver. Participants reported their sociodemographic information, behavioral and medical history via interviewer‐administered questionnaires, and had DXA scans at baseline (1995–1997) and 10 years later (2005–2007). Research ethics boards at McGill University (coordinating center) and at each local institution (including the University of British Columbia's Clinical Research Ethics Board) approved CaM*os* study procedures. All participants provided written informed consent.

From the Vancouver CaM*os* cohort, which included 728 women, controls were selected to match one‐to‐one to the 49 WLWH cases as best possible by age, within 8 years, and by a dichotomized ethnicity variable (White/Indigenous/Black, which included WLWH who self‐identified as either White/Caucasian, Aboriginal/First Nations/Métis/Inuit/Indigenous, or African/Caribbean/Black; or Asian, which included WLWH who self‐identified as South Asian, Southeast Asian, East Asian, or other Asian). We used the dichotomous variable because the Vancouver CaM*os* cohort did not include any participants of Indigenous ancestry and only one participant of Black ancestry. This meant that all Indigenous (*n* = 11) and Black (*n* = 2) WLWH were matched to White controls, whereas all Asian WLWH (*n* = 6) were matched to Asian controls (all of whom were of East Asian ancestry).

### Measurements

Baseline data for WLWH were acquired between May 2001 and September 2003; follow‐up data were acquired between July 2013 and August 2017. At baseline, questionnaires and blood work were completed at Oak Tree Clinic by research staff. Bone imaging took place at BC Women's Hospital + Health Centre within 6 months of the CARMA study visit. At follow‐up, WLWH completed questionnaires and blood work at their CARMA study visit, which took place at the Oak Tree Clinic at BC Women's Hospital + Health Centre. For 18 WLWH, bone imaging took place at BC Women's Hospital + Health Centre, as part of their standard care, within 6 months of their CARMA study visit. For the remaining WLWH (*n* = 31), bone imaging was done on the same day as the study visit, with DXA scans acquired either at BC Women's Hospital + Health Centre (*n* = 25) or at the Centre for Hip Health and Mobility (*n* = 6) providing common‐phantom standardized data. CARMA‐OSTEO data were managed using REDCap (Research Electronic Data Capture)^(^ ^22^, ^23^ ^)^ electronic data capture tools hosted at BC Children's Hospital Research Institute.

Baseline and follow‐up data for controls were acquired at the Vancouver CaM*os* coordinating center at Vancouver General Hospital. At baseline and 10‐year follow‐up, DXA scans were acquired on the same day or within 1 month of the questionnaire data.

### Anthropometry and questionnaires

For all participants, height and weight were measured without shoes and wearing light clothing before the bone imaging, and body mass index (BMI, kg/m^2^) was calculated. Relevant demographic, medication, chronic conditions (eg, hypertension, liver disease, eating disorders), substance use, menopause status (menopausal defined as 12 months without menstrual flow), fracture history, physical activity (regular physical activity yes/no, hours/week of walking, and hours/week doing moderate, strenuous, and vigorous activities), and total calcium and vitamin D intakes (dietary and supplements) were determined using the CaM*os* Questionnaire^(^ ^21^ ^)^ (Year 0 and Year 10 versions) for both WLWH and controls. Additional CARMA‐specific questionnaires (for WLWH only) collected HIV‐specific data. Trained CARMA research staff administered the questionnaires to WLWH, and local CaM*os* personnel administered the CaM*os* questionnaire to controls.

### Clinical and laboratory outcomes

For WLWH, we obtained date of HIV diagnosis, nadir CD4^+^ cell count, peak (highest recorded) HIV plasma viral load (dichotomized as < or ≥100,000 copies/mL), as well as cART duration and regimen type from medical charts. CD4^+^ cell count (absolute and %) and HIV viral load determined as part of standard care closest to and within 6 months of bone imaging were used as current values.

### Bone mass

At baseline, aBMD (g/cm^2^) of the lumbar spine (L_1_ to L_4_) and the nondominant total hip and femoral neck subregion were measured in WLWH using a Hologic (Marlborough, MA, USA) Delphi A densitometer and in controls using a Hologic QDR 1000 densitometer. At follow‐up, aBMD was measured in WLWH using either a Hologic Discovery A (*n* = 43) or Hologic Discovery W (*n* = 6), whereas in controls, year 10 aBMD measurements were obtained using a Lunar Prodigy (GE Healthcare, Madison, WI, USA) densitometer. At each time point, technicians performed daily machine calibration and daily and weekly quality‐assurance tests. At baseline and follow‐up, all data were converted to Hologic values for analysis using standard methods.^(^ ^24^, ^25^ ^)^ Cross‐calibration of DXA instruments was assessed through measurement of the CaM*os* Bona Fide phantom (BFP, Bio‐Imaging Technologies, Newtown, PA, USA) on each unit.^(^ ^26^, ^27^, ^28^ ^)^ For CaM*os*, all densitometers were calibrated at the start of the study and annually thereafter using one Bona Fide Spine Phantom that was sent to each study site in turn. Once the phantom was received at each site, it was scanned 10 times without repositioning. All aBMD data were adjusted to the Bona Fide phantom. The same phantom was scanned on the DXA instruments in Vancouver that were used to measure aBMD in WLWH and controls. Again, all data were adjusted to the common phantom. Short‐term precision (reproducibility) for aBMD was <1.5% at all sites on all scanners.

For WLWH and controls, we calculated 10‐year aBMD change by dividing the aBMD difference (follow‐up minus baseline) by the number of days elapsed between the follow‐up and baseline DXA measurements and then multiplying that result by an equivalent of 10 years in days. We defined the 10‐year aBMD percent change as 100 times the 10‐year aBMD change (above) divided by the baseline aBMD.

### Fracture

For WLWH, fracture history at baseline and follow‐up was reported on the CaM*os* questionnaire. For controls, incident fractures were captured yearly either during in‐person follow‐up interviews (years 5 and 10) or via yearly mailed questionnaires followed by phone interview and, with permission, retrieval of medical documentation. Participants reported number of fractures, fracture site, and type (low, moderate, or high energy). We included low‐moderate trauma (fragility) fractures (ie, those occurring at sites other than the skull, face, hands, or feet without trauma or from less than or equivalent to a fall from standing height) but excluded high‐trauma and pathological fractures due to malignancy. Major osteoporotic fractures were defined as fragility fractures from the hip, spine (clinical), forearm/wrist, or humerus.

### Statistical analysis

As a substudy of CARMA‐CORE, the CARMA‐OSTEO cohort represents a convenience sample and, therefore, we did not perform any sample size calculations. We used SAS Studio release 3.8 (2012–2018, SAS Institute Inc., Cary, NC, USA) for all analyses, and considered *p* < 0.05 statistically significant. Descriptive statistics are presented as number (%) and mean (standard deviation [SD]). Chi‐square (categorical variables) and Student's *t* tests (continuous variables) were used to compare baseline characteristics of WLWH and controls.

Before addressing our primary objective (comparing 10‐year aBMD change between WLWH and controls), we first explored whether aBMD differed between groups at baseline. We fit a series of univariable linear regressions for all baseline characteristics, except for those comorbidities (yes/no categorical variable) where the number of women (WLWH and controls combined) was 5 or less. Variables significantly (*p* < 0.05) associated with baseline aBMD at either L_1_ to L_4_, femoral neck, or total hip were included as covariables in the multivariable linear regression models to compare aBMD between WLWH and controls at baseline. Using this approach, baseline models were adjusted for the following variables: age (with polynomials of up to 3 degrees), height, BMI, race (White/Aboriginal/Other or Asian), employment status (working full‐time, working part‐time, being disabled, other), alcohol consumption (≤1 alcohol serving/d [low] or >1 alcohol serving/day [high]), and menopausal status (pre/peri versus menopausal).

We used a similar modeling strategy to address our primary objective. First, we fit univariable regression models to examine associations between baseline characteristics and BMI change, and percent change in L_1_ to L_4_, total hip, and femoral neck aBMD. Only variables significantly associated with either L_1_ to L_4_, total hip, or femoral neck aBMD percent change were included in the multivariable linear regressions. The final three models for aBMD percent change were adjusted for the following baseline variables: age, height, BMI, alcohol consumption, menopausal status, and hypertension. We explored the potential influence of a group x change in menopausal status (ie, became menopausal during follow‐up versus remained premenopausal at follow‐up) interaction. However, because of collinearity between age and becoming menopausal at follow‐up (*r* = 0.8), we did not include the interaction term in the final model.

To address our secondary objective (associations between HIV clinical characteristics and aBMD [baseline and change]), we fit a series of multivariable regression models for each aBMD site. We included, in turn, each of the baseline HIV clinical characteristics, adjusted for baseline age, height, and BMI. Again, polynomials of up to 3 degrees were considered for age. We considered plasma HIV viral load as detectable if >40 copies/mL. Because both viral load measures, at baseline and at follow‐up, were not normally distributed within WLWH with detectable levels, they were log‐transformed.

## Results

Of the 76 CWHS participants originally enrolled at the Vancouver site between 2001 and 2003, 53 WLWH were still receiving care at the Oak Tree Clinic between 2013 and 2017. Among these, 51 were enrolled in CARMA‐OSTEO and 50 completed the follow‐up study visit; one woman died during the follow‐up period. One WLWH did not complete the CaM*os* questionnaire at baseline and was excluded from this analysis. Therefore, this analysis includes 49 WLWH with DXA scans at both baseline and follow‐up.

For WLWH, follow‐up data (questionnaire and DXA scans) was acquired on average 13.2 years (range 10 to 15 years) after the baseline data. The duration of follow‐up was shorter for controls, with follow‐up data collected on average 10.1 years (range 9.9 to 10.5 years) after baseline data. Baseline characteristics for WLWH and controls are shown in Table 1. Compared with controls, WLWH were, on average, 5 years younger, more were pre/perimenopausal, and fewer had completed high school or were employed full‐time. More than 40% of the WLWH were on disability, whereas none were in the control group. Calcium and vitamin D supplement use was higher among WLWH compared with controls, as was prevalence of liver disease (46% versus 6%) and eating disorders (10% versus 0%). Prevalence of inflammatory bowel disease was lower among WLWH than controls (0% versus 8%). A similar proportion of WLWH (37%) and controls (39%) transitioned to menopause during the follow‐up period.

**Table 1:** Baseline Characteristics for Women Living With HIV (WLWH) in the CARMA‐OSTEO Cohort and for HIV‐Negative Women in the Vancouver CaM*os* Cohort (Controls)

		WLWH (*n* = 49)	Controls (*n* = 49)	Mean difference or difference in proportions (95% CI)	*p* Value^a^
Age (years), mean (SD)		36.8 (8.8)	41.9 (9.2)	**−5.1 (−8.7; −1.5)**	0.006
Race	White, Black or Indigenous	43 (87.8%)	43 (87.8%)	0.0% (−0.1; 0.1)	>0.99
	Asian	6 (12.2%)	6 (12.2%)	—	
Education	More than high school	26 (53.0%)	37 (75.5%)	**−22.5% (−40.9; 4.0)**	0.017
Employment	Disability	21 (42.9%)	0 (0.0%)	**42.9% (29.0; 56.7)**	<0.001
Live alone		13 (26.5%)	7 (14.3%)	12.2% (−3.5; 28.0)	0.128
Height (cm), mean (SD)		163.0 (7.6)	162.3 (7.3)	0.7 (−2.3; 3.7)	0.647
BMI (kg/m^2^), mean (SD)		24.6 (5.4)	25.3 (5.3)	−0.7 (−2.8; 1.5)	0.531
10‐year body mass index change (kg/m^2^)		0.8 (3.2)	1.6 (2.2)	−0.8 (−1.9; 0.3)	0.163
Ever use of combined hormonal contraceptives		41 (83.7%)	39 (79.6%)	4.1% (−11.2; 19.4)	0.601
Menopausal women		2 (4.1%)	10 (20.4%)	**−16.3% (−28.9; −3.8)**	0.011
No. of pregnancies, mean (SD)		2.0 (1.4)	1.8 (1.5)	0.1 (−0.4; 0.7)	0.626
No. of live births, mean (SD)		1.1 (1.1)	1.3 (1.1)	−0.2 (−0.6; 0.3)	0.404
Alcohol intake	None	25 (54.4%)	17 (34.7%)	19.7% (−0.0; 39.3)	0.050
Smoking	Never	18 (37.5%)	34 (69.4%)	**−31.9% (−50.7; −13.1)**	<0.001
Participating in regular physical activity		29 (59.2%)	29 (59.2%)	0.0% (−19.5; 19.5)	>0.99
No. of hours walking/week, mean (SD)		6.5 (5.8)	4.1 (3.4)	**2.3 (0.4; 4.2)**	0.018
No. of hours moderate, strenuous & vigorous activities/week, mean (SD)		19.6 (10.4)	16.6 (11.6)	3.1 (−1.3; 7.5)	0.170
Calcium supplement use		40 (81.6%)	19 (38.8%)	**42.9% (25.4; 60.3)**	<0.001
Vitamin D supplement use		39 (79.6%)	12 (24.5%)	**55.1% (38.6; 71.6)**	<0.001
Dietary calcium intake (mg/d), mean (SD)		1031 (762)	790 (445)	241 (−23; 504)	0.073
Dietary vitamin D intake (IU/d), mean (SD)		96 (132)	88 (100)	6 (−41; 54)	0.790

*Note*: Values are presented as count (%) and mean difference (95% confidence interval [CI]). Bold text indicates a significant difference between WLWH and controls.

^a^ *t* tests were used to test mean differences between WLWH and controls (pooled or Satterthwaite approximation depending on equal or unequal variances); a two‐sample *Z* test was used to test the equality of two proportions.

The clinical characteristics of WLWH at baseline and follow‐up are presented in Table 2. Among WLWH, 35% had an undetectable viral load at baseline, and this increased to 80% at follow‐up. Nine women started cART after baseline; the average time between their baseline DXA scan and cART start was 5.5 years (range 2.3 to 7.9 years). One WLWH remained cART‐naïve at follow‐up. Whereas no WLWH received tenofovir disoproxil fumarate (tenofovir) at baseline, 86% of WLWH received tenofovir during follow‐up.

**Table 2:** Clinical Characteristics of Women Living With HIV in the CARMA‐OSTEO Cohort

	Baseline			Follow‐up		
	*n* (%)	Median (IQR)	Range	*n* (%)	Median (IQR)	Range
Age (years) at HIV diagnosis	48 (98%)	29.0 (25.0–36.6)	17.0–49.4	—	—	—
Time since HIV diagnosis (years), *n* = 48	48 (98%)	6.6 (3.4–8.9)	0.8–14.4	—	—	—
Age (years) at CD4 nadir	44 (90%)	35.5 (27.5–40.8)	18.0–51.3	—	—	—
CD4 nadir (cells/μL)	44 (90%)	260 (100–360)	1–1000	35 (71%)	140 (50–250)	0–630
CD4 nadir (%)	44 (90%)	22 (12–28)	2–40	35 (71%)	14 (10–20)	2–53
CD4 count (cells/μL)	44 (90%)	390 (250–620)	30–2380	35 (71%)	545 (320, 810)	150–2380
CD4 count (%)	44 (90%)	28 (16–34)	4–52	35 (71%)	31 (26, 41)	7–62
Peak HIV pVL ≥100,000 copies/mL	26 (53%)	—	—	—	—	—
Log HIV pVL (copies/ mL), *n* = 25	25 (51%)	10.2 (8.9–11.2)	7.7–12.3	7 (14%)	4.5 (4.0, 4.6)	3.8–4.8
HIV pVL undetectable (<40 copies/mL)	24 (49%)	—	—	42 (86%)	—	—
On cART >3 months	36 (80%)	—	—	48 (98%)	—	—
Lifetime cART duration (weeks)	36 (80%)	231 (147–324)	18–635	48 (98%)	739 (506–935)	61–1304
TDF use (ever)	0 (0.0%)	—	—	42 (86%)	—	—
Duration of TDF use (weeks)	0 (0.0%)	—	—	42 (86%)	325 (198–503)	1–612

Abbreviations: cART = combination antiretroviral therapy TDF = tenofovir disoproxil fumarate; IQR = interquartile range; pVL = plasma viral load.

*Note*: In WLWH with detectable plasma viral load.

### Baseline aBMD and 10‐year change in aBMD

Table 3 presents baseline and 10‐year percent change values for our primary outcomes of L_1_ to L_4_, total hip, and femoral neck aBMD in WLWH and controls. At baseline, aBMD was not significantly different between WLWH and controls at any site. For 10‐year percent change, WLWH experienced significant declines in aBMD at all three sites, whereas controls demonstrated significant declines in total hip and femoral neck aBMD but not L_1_ to L_4_ aBMD. The magnitude of aBMD decline did not differ significantly between WLWH and controls, and a similar proportion of WLWH and controls demonstrated greater than 5% loss in aBMD over the 10 years at L_1_ to L_4_ (40% versus 35%), total hip (49% versus 45%), and femoral neck (69% versus 55%). However, we note that the confidence intervals for aBMD 10‐year percent change did not exclude clinically important differences. In addition, two controls were apparent outliers for aBMD percent change, one at L_1_ to L_4_ and the other at the total hip and femoral neck sites. We performed a sensitivity analysis to determine if these individuals influenced the results. Estimates changed slightly for adjusted percent change in L_1_ to L_4_ aBMD (−1.39 [−4.14; 1.36]), total hip aBMD (−2.85 [−5.86; 0.16]), and femoral neck aBMD (−2.12 [−4.84; 0.60]), but remained not statistically significant. We performed a second sensitivity analysis that included only WLWH and controls who self‐identified as White or Caucasian. Estimates changed slightly for adjusted 10‐year percent change in aBMD at each site, but group differences remained not statistically significant (Supplemental Table S1).

**Table 3:** Baseline and 10‐Year Percent Change in Areal Bone Mineral Density (aBMD) at the Lumbar Spine (L_1_ to L_4_), Total Hip, and Femoral Neck Subregions in Women Living with HIV (WLWH) in the CARMA‐OSTEO Cohort and HIV‐Negative Women in the Vancouver CaM*os* Cohort (Controls)

		WLWH (*n* = 49)	Controls (*n* = 49)	Unadjusted difference	Adjusted^a^ difference
		Mean ± SD (95% CI)	Mean ± SD (95% CI)	Estimate (95% CI)	Estimate (95% CI)
L_1_ to L_4_	Baseline (g/cm^2^)	0.996 ± 0.114 (0.964; 1.029)	1.024 ± 0.119 (0.989; 1.058)	−0.027 (−0.074; 0.020)	−0.018 (−0.074; 0.039)
	10‐year change (g/cm^2^)	−0.035 ± 0.069^b^ (−0.055; −0.015)	−0.028 ± 0.076 (−0.050; −0.006)	—	—
	10‐year % change	**−3.25 ± 6.98** ^b^ **(−5.27; −1.22)**	−2.36 ± 8.37 (−4.76; 0.04)	−0.89 (−4.00; 2.22)	−1.69 (−4.85; 1.47)
Total hip	Baseline (g/cm^2^)	0.890 ± 0.122 (0.855; 0.925)	0.922 ± 0.121 (0.887; 0.957)	−0.032 (−0.081; 0.016)	−0.020 (−0.075; 0.034)
	10‐year change (g/cm^2^)	**−0.043 ± 0.053 (−0.058; −0.028)**	**−0.035 ± 0.069 (−0.054; −0.015)**	—	—
	10‐year % change	**−4.57 ± 6.24 (−6.36; −2.78)**	**−3.47 ± 7.59 (−5.65; −1.29)**	−1.10 (−3.89; 1.69)	−1.55 (−4.51; 1.41)
Femoral neck	Baseline (g/cm^2^)	0.772 ± 0.114^b^ (0.739; 0.805)	0.774 ± 0.106 (0.743; 0.804)	−0.002 (−0.046; 0.042)	0.002 (−0.045; 0.049)
	10‐year change (g/cm^2^)	**−0.053 ± 0.065** ^b^ **(−0.072; −0.035)**	**−0.043 ± 0.063 (−0.061; −0.025)**	—	—
	10‐year % change	**−6.53 ± 8.06** ^b^ **(−8.87; −4.19)**	**−5.40 ± 8.58 (−7.87; −2.94)**	−1.13 (−4.49; 2.22)	−2.05 (−5.50; 1.41)

*Note*: Bold text indicates change values are significantly different from 0 as determined using a two‐sample *t* test.

^a^ Baseline aBMD models were adjusted for baseline age (polynomials of up to 3 degrees considered), height, body mass index (BMI), race, employment status, alcohol consumption, and menopausal status; 10‐year aBMD percent change models were adjusted for the following baseline variables: age (polynomials of up to 3 degrees considered), height, BMI, alcohol consumption, menopausal status, and hypertension.

^b^ *n* = 48.

### Associations between clinical characteristics and aBMD in WLWH

Associations between clinical characteristics and aBMD (baseline and follow‐up) in WLWH are presented in Supplemental Tables S2 and S3. When considering baseline aBMD, only total duration of cART was significantly associated with femoral neck aBMD. However, the negative association was no longer significant after adjusting for age, height, and BMI, but still did not exclude clinically important differences. When considering 10‐year percent change in aBMD, baseline CD4 count was positively associated with femoral neck aBMD percent change before and after adjusting for covariates. Being on cART for more than 3 months before baseline was negatively associated with L_1_ to L_4_ aBMD percent change, but this association was not significant after adjusting for covariates. Becoming menopausal during follow‐up was negatively associated with aBMD percent change at all three sites in unadjusted analyses only. Finally, an increase in BMI across 10 years was positively associated with total hip aBMD 10‐year percent change before and after adjusting for covariates. Other HIV clinical characteristics, including use of tenofovir, were not associated with aBMD change at any site.

### Fracture history

At baseline, more WLWH had experienced prevalent low‐trauma and major osteoporotic fractures compared with controls (Table 4). During follow‐up, the number of women who sustained an incident fracture (low trauma and major osteoporotic) did not differ significantly between groups (Table 4). However, there was a trend for more WLWH to experience an incident fragility fracture than controls (10% versus 2%). In a sensitivity analysis that included only WLWH and controls who self‐identified as White or Caucasian, results were similar to those of the full cohort for prevalent fragility fractures at baseline. However, the group difference in prevalent major osteoporotic fractures at baseline was no longer statistically significant. We provide these results in Supplemental Table S4.

**Table 4:** Number (%) of Women Living With HIV (WLWH) and HIV‐Negative Controls Who Reported Fractures at Baseline and Follow‐Up

Fracture type		WLWH (*n* = 49)	Controls (*n* = 49)	Difference in proportion (95% CI)
Prevalent (baseline)	Any fragility fracture excluding hand, foot, skull	14 (29.8%)	5 (10.2%)	**19.6% (4.0; 35.2)**
	Major osteoporotic fracture	8 (17.0%)	2 (4.1%)	**12.9% (0.9; 25.0)**
Incident (follow‐up period)	Any fragility fracture excluding hand, foot, skull	5 (10.4%)	1 (2.0%)	8.4% (−1.1; 17.9)
	Major osteoporotic fracture	4 (8.3%)	1 (2.0%)	6.3% (−2.5; 15.1)

*Note*: Bold text indicates a significant difference between WLWH and controls.

Abbreviations: Major osteoporotic fracture = fragility fracture of the hip, forearm/wrist, clinical spine, or humerus/shoulder fracture.

## Discussion

In this prospective observational study, we contribute novel data on change in aBMD across 10 years in pre/peri‐ and menopausal women living with HIV in Vancouver, Canada. When compared with similar‐aged women without HIV living in the same geographic area, WLWH, 98% of whom had ever received cART, did not differ with respect to either aBMD at study entry or the trajectory of bone loss during follow‐up. However, given the small sample size and the observation that confidence intervals for 10‐year percent change did not exclude clinically important differences, we acknowledge that further study is warranted to confirm this finding. Overall, HIV‐related clinical characteristics were not associated with 10‐year percent change in aBMD in WLWH, although WLWH with better controlled HIV may experience reduced aBMD loss at the femoral neck. Given our small sample, it was also not possible to accurately compare incident fractures between WLWH and controls; however, a possible trend for higher fracture incidence among WLWH requires further investigation, particularly in later adulthood.

### Baseline and 10‐year change in aBMD

Similar to our previous study of WLWH^(^ ^12^ ^)^ (which included the 49 women in the present analysis), baseline aBMD did not differ between WLWH and controls at any site, yet numerically more WLWH had a history of low‐trauma fracture and major osteoporotic fracture compared with controls. In our previous study, which included a larger proportion of Black WLWH (16.5% versus 1% of controls) than the current study (4% Black WLWH versus 0 controls), ethnic differences in aBMD may have skewed the average aBMD values.^(^ ^12^ ^)^ However, we recently reported significant deficits in distal radius and tibia volumetric BMD, trabecular bone microarchitecture, and estimated bone strength as measured with HR‐pQCT in the current WLWH cohort (measurements taken at the follow‐up visit for the present study).^(^ ^20^ ^)^ These deficits persisted after adjusting for osteoporosis risk factors. Although we do not have similar HR‐pQCT data at baseline of the present study, it is possible that BMD, bone microarchitecture, and strength may have been compromised in WLWH at that time and may have contributed to their higher fracture prevalence. As more WLWH reach older adulthood, longitudinal studies of bone microarchitecture and strength are clearly needed to enhance our understanding of how these characteristics of bone health influence fracture risk in WLWH.

To our knowledge, this is the longest follow‐up study of aBMD in WLWH that also includes a geographical, randomly sampled comparison group of women without HIV. It is, therefore, difficult to interpret our findings in the context of previous prospective studies of WLWH.^(^ ^13^, ^17^, ^29^, ^30^, ^31^, ^32^, ^33^ ^)^ We can look, however, to the 6‐year longitudinal study of Bolland and colleagues that included 44 men living with HIV (MLWH, aged 49 years at baseline) who had all received cART for at least 3 months at baseline, and 37 healthy controls (aged 46 years at baseline).^(^ ^34^ ^)^ Across 6 years, aBMD change did not differ between MLWH and controls at either the total hip or total body. At the lumbar spine, MLWH had a greater increase in aBMD compared with controls, which may have been due, in part, to greater gains in lean body mass.^(^ ^34^ ^)^ Stability in aBMD was observed in MLWH despite the presence of key osteoporosis risk factors, including lower body weight and higher smoking prevalence. Together with results from our longitudinal study and from previous short‐term longitudinal studies,^(^ ^13^, ^17^, ^29^, ^30^, ^31^, ^32^, ^33^ ^)^ these findings suggest that HIV status is not independently associated with accelerated bone loss as assessed by DXA in PLWH with suppressed viral load. It is possible that changes in clinical HIV care, including limiting cART interruptions, starting cART as soon as possible after HIV diagnosis, and incorporating regular calcium and vitamin D supplementation, may contribute to maintaining bone health in WLWH. In this context, results of the present study support consensus statements that routine DXA screening for all PLWH is not recommended before 50 years of age.^(^ ^35^ ^)^ However, as we acknowledge above, further investigation in a larger cohort is warranted to confirm our findings. Further, this does not preclude situations where targeted screening may be appropriate such as in WLWH who are at risk for hypothalamic amenorrhea.^(^ ^36^ ^)^

Given our relatively small sample size, we were also unable to accurately assess potential differences in fracture incidence during follow‐up between WLWH and controls. The possible trend for higher fracture rates in WLWH we observed aligns with current evidence that HIV infection is associated with moderately greater risk for incident fracture.^(^ ^9^ ^)^ However, there is a need for additional prospective studies to confirm this increased risk and to determine if risk factors for fracture in WLWH differ from those in women living without HIV.

### Potential determinants of aBMD change in WLWH

Across a number of HIV‐related clinical characteristics, only baseline CD4 count was significantly associated with aBMD change in WLWH; this positive association was only apparent at the femoral neck. Similar observations were reported in longitudinal studies of PLWH after cART initiation.^(^ ^37^, ^38^ ^)^ These findings support the potential role for T‐cell lymphocytes in maintaining bone health. Specifically, within the emerging field of osteoimmunology or “immunoporosis,”^(^ ^39^ ^)^ direct and indirect effects of T cells on osteoclastogenic function and bone remodeling have been reported.^(^ ^39^ ^)^ In addition, spinal aBMD loss in WLWH has been related to accelerated aging as shown by shorter leukocyte telomere lengths.^(^ ^40^ ^)^

Similar to previous longitudinal studies,^(^ ^32^, ^33^ ^)^ the duration of cART was not associated with aBMD change at any site. However, in the absence of more frequent aBMD measurements during follow‐up, we were unable to determine how aBMD changed in the 8 women who started cART (and tenofovir more specifically) after the baseline study visit. If these women experienced accelerated aBMD loss in the first 2 years after starting cART similar to that reported in previous studies,^(^ ^13^, ^32^ ^)^ it appears that aBMD recovered and the rate of change stabilized thereafter.

Of the non‐HIV‐related factors, only 10‐year BMI change was significantly associated with 10‐year percent change in aBMD before and after adjusting for covariates, and this relationship was evident only at the total hip. We were unable to assess body composition in this study and, therefore, cannot determine whether this association was mediated by changes in lean or fat mass, both of which are influenced by HIV‐related clinical characteristics^(^ ^41^, ^42^, ^43^ ^)^ and aging.^(^ ^44^ ^)^ Previous studies of PLWH reported positive associations between change in total hip aBMD and change in both lean and fat mass.^(^ ^45^, ^46^ ^)^ Given the influence of body composition, and particularly lean mass, on healthy aging,^(^ ^47^ ^)^ future longitudinal studies of WLWH would benefit from assessing changes in lean and fat mass.

### Limitations

We acknowledge several limitations of our study. First, although we attempted to match WLWH and controls according to age and ethnic origin, demographic differences between the two cohorts prevented us from doing so. Compared with controls, our small cohort of WLWH included more Indigenous (22%) and Black (4%) women, which was representative of the PLWH population in Metro Vancouver at the time of the study.^(^ ^48^ ^)^ As the control CaM*os* cohort did not include any Indigenous or Black women in this age range, we grouped these women with White WLWH. Ethnic differences in aBMD between Black and White women are well established,^(^ ^49^ ^)^ but to our knowledge, only one study has examined aBMD in Indigenous Canadian women. In the First Nations Bone Health study, lower weight‐adjusted aBMD in Aboriginal (sic) women was attributed to a lower ratio of lean mass to fat mass compared with White women.^(^ ^50^ ^)^ This further highlights the need to incorporate measures of body composition into future studies of WLWH, particularly in ethnically diverse cohorts. Second, given our relatively small convenience sample of WLWH, we did not have sufficient power to examine changes in aBMD according to menopausal status. Third and finally, we were unable to consider the influence of hepatitis C virus infection (for WLWH or controls) in our analyses because of data unavailability.

In summary, our findings in this small cohort of WLWH under care suggest that trajectories of aBMD mirror those of normal aging, at least in mid‐adulthood. Further study is warranted to understand how aBMD and three‐dimensional aspects of bone microarchitecture and bone strength change as WLWH age and transition to menopause, and how such changes, if any, may contribute to fracture risk in this population. Despite the small sample of WLWH, it is reassuring that bone loss was not pathologically accelerated in WLWH during mid‐adulthood and also that the reported rapid loss of aBMD when starting cART did not persist.

## Author Contributions

**Heather M. Macdonald:** Formal analysis; methodology; validation; visualization; writing – original draft; writing – review and editing. **Evelyn J. Maan:** Conceptualization; data curation; funding acquisition; investigation; methodology; project administration; resources; writing – review and editing. **Claudie Berger:** Data curation; formal analysis; methodology; validation; visualization; writing – original draft; writing – review and editing. **Helene C. F. Cote:** Conceptualization; funding acquisition; investigation; methodology; project administration; resources; supervision; writing – review and editing. **Melanie C. M. Murray:** Conceptualization; funding acquisition; investigation; methodology; project administration; resources; supervision; writing – review and editing. **Neora Pick:** Conceptualization; funding acquisition; investigation; project administration; resources; supervision; writing – review and editing. **Jerilynn C. Prior:** Conceptualization; funding acquisition; investigation; supervision; writing – review and editing.

## Disclosures

All authors state that they have no conflicts of interest.

### Peer Review

The peer review history for this article is available at https://www.webofscience.com/api/gateway/wos/peer-review/10.1002/jbm4.10761.

## Supporting information


**Table S1.** Baseline and 10‐year percent change in areal bone mineral density (aBMD) at the lumbar spine (L_1_ to L_4_), total hip, and femoral neck subregions in White women living with HIV (WLWH) in the CARMA‐OSTEO cohort and White HIV‐negative women in the Vancouver CaM*os* cohort (Controls). Bold text indicates change values are significantly different from zero as determined using a two‐sample *t* test.
**Table S2.** Estimates and 95% confidence intervals in women living with HIV for each of the clinical characteristics of interest, adjusted for baseline age, height, and BMI for baseline areal bone mineral density (aBMD) at the lumbar spine (L_1_ to L_4_), total hip, and femoral neck subregions.
**Table S3.** Estimates and 95% confidence intervals in women living with HIV for each of the clinical characteristics of interest, adjusted for baseline age, height, and BMI for 10‐year percent change in areal bone mineral density (aBMD) at the lumbar spine (L_1_ to L_4_), total hip, and femoral neck subregions.
**Table S4.** Number (%) of White women living with HIV (WLWH) and White HIV‐negative controls who reported fractures at baseline and follow‐up. Bold text indicates a significant difference between White WLWH and controls.
