# Supplementary material for: Long‐Term Change in Bone Mineral Density in Women Living With HIV: A 10‐Year Prospective Controlled Cohort Study
Source: JBMR Plus. 2023 Jul 5;7(8):e10761. doi: 10.1002/jbm4.10761 (PMC10443077; doi:10.1002/jbm4.10761)
Supplement: Supplementary file 1 — Table S1. Baseline and 10‐year percent change in areal bone mineral density (aBMD) at the lumbar spine (L1 to L4), total hip, and femoral neck subregions in White women living with HIV (WLWH) in the CARMA‐OSTEO cohort and White HIV‐negative women in the Vancouver CaMos cohort (Controls). Bold text indicates change values are significantly different from zero as determined using a two‐sample t test. Table S2. Estimates and 95% confidence intervals in women living with HIV for each of the clinical characteristics of interest, adjusted for baseline age, height, and BMI for baseline areal bone mineral density (aBMD) at the lumbar spine (L1 to L4), total hip, and femoral neck subregions. Table S3. Estimates and 95% confidence intervals in women living with HIV for each of the clinical characteristics of interest, adjusted for baseline age, height, and BMI for 10‐year percent change in areal bone mineral density (aBMD) at the lumbar spine (L1 to L4), total hip, and femoral neck subregions. Table S4. Number (%) of White women living with HIV (WLWH) and White HIV‐negative controls who reported fractures at baseline and follow‐up. Bold text indicates a significant difference between White WLWH and controls. [file JBM4-7-e10761-s001.docx]

**Supplementary Table 1.** Baseline and 10-year percent change in areal bone mineral density (aBMD) at the lumbar spine (L1-L4), total hip and femoral neck subregions in White women living with HIV (WLWH) in the CARMA-OSTEO cohort and White HIV-negative women in the Vancouver CaM*os* cohort (Controls). Bold text indicates change values are significantly different from zero as determined using a two-sample t-test.

|  | | **WLWH**  **(n=29)** | **Controls**  **(n=29)** | **Unadjusted**  **Difference** | **Adjusted^**  **Difference** |
| --- | --- | --- | --- | --- | --- |
|  | | **Mean ±SD**  **(95% CI)** | **Mean ±SD**  **(95% CI)** | **Estimate (95% CI)** | **Estimate (95% CI)** |
| L1-L4 | Baseline (g/cm^2^) | 1.023 ± 0.106  (0.983; 1.063) | 1.023 ± 0.109  (0.981; 1.064) | 0.000 (-0.056; 0.057) | 0.023 (-0.051; 0.097) |
|  | 10-yr change (g/cm^2^) | **-0.036 ± 0.069**  **(-0.062; -0.009)** | **-0.044 ± 0.073**  **(-0.072; -0.017)** | ------ | ------ |
|  | 10-yr % change | **-3.29 ± 6.97**  **(-5.94; -0.64)** | **-4.19 ± 7.20**  **(-6.93; -1.46)** | 0.90 (-2.83; 4.63) | -1.00 (-4.94; 2.95) |
| Total hip | Baseline (g/cm^2^) | 0.887 ± 0.123  (0.840; 0.934) | 0.918 ± 0.129  (0.869; 0.967) | -0.031 (-0.097; 0.035) | -0.007 (-0.084; 0.070) |
|  | 10-yr change (g/cm^2^) | **-0.053 ± 0.048**  **(-0.071; -0.034)** | **-0.036 ± 0.071**  **(-0.063; -0.008)** | ------ | ------ |
|  | 10-yr % change | **-5.75 ± 6.15**  **(-8.09; -3.41)** | **-3.43 ±7.23**  **(-6.19; -0.69)** | -2.31 (-5.84; 1.22) | -2.07 (-6.03; 1.88) |
| Femoral neck | Baseline (g/cm^2^) | 0.762 ± 0.118  (0.717; 0.807) | 0.769 ± 0.100  (0.730; 0.807) | -0.006 (-0.064; 0.051) | 0.003 (-0.062; 0.068) |
|  | 10-yr change (g/cm^2^) | **-0.048 ±0.063**  **(-0.072; -0.024)** | **-0.037 ± 0.059**  **(-0.059; -0.015)** | ------ | ------ |
|  | 10-yr % change | **-6.02 ± 8.16**  **(-9.13; -2.92)** | **-4.43 ± 7.41**  **(-7.24; -1.61)** | -1.60 (-5.70; 2.50) | -2.12 (-5.64; 1.40) |

^Baseline aBMD models were adjusted for baseline age (polynomials of up to 3 degrees considered), height, BMI, race, employment status, alcohol consumption and menopausal status; Ten-year aBMD percent change models were adjusted for the following baseline variables: age (polynomials of up to 3 degrees considered), height, BMI, alcohol consumption, menopausal status and hypertension.

**Supplemental Table 2.** Estimates and 95% confidence intervals in women living with HIV for each of the clinical characteristics of interest, adjusted for baseline age, height and BMI for baseline areal bone mineral density (aBMD) at the lumbar spine (L1-L4), total hip and femoral neck subregions.

|  | **L1-L4 aBMD** | | **Total hip aBMD** | | **Femoral neck aBMD** | |
| --- | --- | --- | --- | --- | --- | --- |
| **Baseline characteristics** | **Unadjusted** | **Adjusted^** | **Unadjusted** | **Adjusted^** | **Unadjusted** | **Adjusted^** |
| Time since HIV diagnosis (years) | 0.002  (-0.007; 0.012) | -0.002  (-0.011; 0.007) | 0.005  (-0.005; 0.015) | 0.008  (-0.001; 0.017) | 0.000  (-0.009; 0.010) | 0.003  (-0.006; 0.011) |
| CD4 nadir (per 1 SD=211 cells/µL) | 0.002  (-0.031; 0.036) | 0.014  (-0.019; 0.047) | -0.004  (-0.040; 0.031) | -0.022  (-0.056; 0.011) | 0.002  (-0.032; 0.036) | -0.018  (-0.049; 0.014) |
| CD4 nadir (%) | 0.001  (-0.002; 0.004) | 0.002  (-0.001; 0.005) | 0.000  (-0.004; 0.003) | -0.001  (-0.004; 0.003) | 0.001  (-0.003; 0.004) | 0.000  (-0.003; 0.003) |
| CD4 count (per 1 SD=358 cells/µL) | -0.019  (-0.052; 0.014) | -0.005  (-0.036; 0.025) | -0.033  (-0.068; 0.001) | -0.029  (-0.059; 0.002) | -0.029  (-0.062; 0.004) | -0.026  (-0.054; 0.003) |
| HIV pVL > 100,000 (Yes vs. No) | -0.031  (-0.152; 0.090) | -0.023  (-0.129; 0.083) | 0.026  (-0.104; 0.155) | 0.024  (-0.087; 0.134) | 0.013  (-0.125; 0.151) | 0.019  (-0.095; 0.133) |
| Peak HIV pVL > 100,000 (Yes vs. No) | 0.020  (-0.046; 0.086) | -0.002  (-0.064; 0.061) | 0.005  (-0.066; 0.076) | 0.006  (-0.059; 0.071) | -0.023  (-0.090; 0.043) | -0.020  (-0.080; 0.039) |
| Log HIV pVL* | -0.003  (-0.016; 0.011) | -0.002  (-0.016; 0.011) | 0.007  (-0.009; 0.022) | 0.008  (-0.007; 0.023) | 0.004  (-0.011; 0.018) | 0.005  (-0.009; 0.018) |
| Log Peak HIV pVL* | 0.007  (-0.015; 0.029) | 0.002  (-0.019; 0.023) | 0.000  (-0.024; 0.023) | 0.004  (-0.018; 0.026) | -0.008  (-0.031; 0.014) | -0.003  (-0.023; 0.017) |
| Prior cART use (>3months: Yes vs. no) | 0.030  (-0.047; 0.108) | 0.022  (-0.058; 0.103) | -0.012  (-0.095; 0.071) | 0.042  (-0.040; 0.125) | -0.022  (-0.101; 0.057) | 0.034  (-0.042; 0.110) |
| cART duration >3 months (per 1 SD=142 weeks) | -0.011  (-0.051; 0.029) | -0.017  (-0.060; 0.026) | -0.024  (-0.066; 0.018) | -0.008  (-0.050; 0.035) | **-0.039**  **(-0.075; -0.003)** | -0.031  (-0.070; 0.008) |
| Liver disease (Yes vs No) | -0.061  (-0.124; 0.003) | -0.049  (-0.107; 0.009) | -0.044  (-0.116; 0.027) | -0.038  (-0.099; 0.023) | -0.005  (-0.074; 0.063) | 0.000  (-0.056; 0.057) |

*In WLWH with detectable HIV plasma viral load (pVL) or peak HIV pVL (i.e., > 40).

^Models adjusted for baseline values of age, height and BMI.

**Supplemental Table 3.** Estimates and 95% confidence intervals in women living with HIV for each of the clinical characteristics of interest, adjusted for baseline age, height and BMI for 10-year percent change in areal bone mineral density (aBMD) at the lumbar spine (L1-L4), total hip and femoral neck subregions.

|  | **L1-L4 aBMD**  **% 10-Year change** | | **Total hip aBMD**  **% 10-Year change** | | **Femoral neck aBMD**  **% 10-Year change** | |
| --- | --- | --- | --- | --- | --- | --- |
|  | **Unadjusted** | **Adjusted^** | **Unadjusted** | **Adjusted^** | **Unadjusted** | **Adjusted^** |
| Time since HIV diagnosis (years) | -0.36  (-0.92; 0.21) | -0.16  (-0.76; 0.44) | -0.37  (-0.86; 0.11) | -0.18  (-0.72; 0.36) | -0.32  (-0.97; 0.32) | -0.27  (-0.96; 0.43) |
| CD4 nadir at baseline (per 1 SD=211 cells/µL) | 0.58  (-1.52; 2.69) | -0.51  (-2.77; 1.75) | 0.17  (-1.66; 2.00) | -0.88  (-2.90; 1.14) | 1.03  (-1.35; 3.40) | 0.44  (-2.29; 3.18) |
| CD4 nadir at baseline (%) | -0.05  (-0.25; 0.15) | -0.13  (-0.34; 0.07) | -0.01  (-0.19; 0.17) | -0.10  (-0.29; 0.08) | 0.08  (-0.16; 0.32) | -0.01  (-0.26; 0.24) |
| CD4 count at baseline  (per 1 SD=358 cells/µL) | 0.30  (-1.75; 2.36) | 0.11  (-1.96; 2.18) | 0.87  (-0.95; 2.68) | 0.62  (-1.26; 2.50) | **2.63**  **(0.35; 4.91)** | **2.59**  **(0.17; 5.02)** |
| CD4 nadir at follow-up  (per 1 SD=130 cells/µL) | -0.32  (-2.39; 1.74) | -1.34  (-3.43; 0.75) | -0.79  (-2.61; 1.03) | -1.70  (-3.58; 0.18) | -0.82  (-3.20; 1.57) | -1.20  (-3.91; 1.50) |
| CD4 nadir at follow-up (%) | -0.14  (-0.37; 0.09) | -0.15  (-0.37; 0.06) | -0.08  (-0.28; 0.13) | -0.11  (-0.29; 0.06) | -0.12  (-0.44; 0.19) | -0.04  (-0.34; 0.26) |
| HIV pVL > 100,000 (Yes vs. No) | 1.74  (-5.66; 9.14) | 1.08  (-6.08; 8.24) | -1.40  (-8.01; 5.20) | -1.85  (-8.39; 4.69) | -0.49  (-10.26; 9.29) | -0.56  (-9.68; 8.57) |
| Peak HIV pVL > 100,000 (Yes vs. No) | 1.12  (-2.97; 5.21) | 2.06  (-2.13; 6.26) | 0.86  (-2.76; 4.49) | 2.02  (-1.79; 5.83) | 3.56  (-1.06; 8.18) | 3.54  (-1.17; 8.25) |
| Log HIV pVL* | -0.19  (-0.98; 0.61) | -0.03  (-0.87; 0.82) | -0.48  (-1.29; 0.33) | -0.43  (-1.27; 0.42) | -0.56  (-1.62; 0.51) | -0.07  (-1.13; 1.00) |
| Log Peak HIV pVL* | 0.03  (-1.23; 1.30) | 0.31  (-1.03; 1.64) | 0.14  (-1.04; 1.32) | 0.53  (-0.73; 1.79) | 0.73  (-0.85; 2.31) | 0.85  (-0.77; 2.48) |
| HIV pVL at follow-up  (detectable vs. undetectable) | -1.32  (-6.35; 3.72) | -2.42  (-7.64; 2.79) | 0.13  (-4.36; 4.63) | -1.13  (-5.94; 3.67) | 0.00  (-5.83; 5.82) | 2.67  (-3.50; 8.85) |
| Peak HIV pVL at follow-up > 100,000  (yes vs. no) | -2.26  (-6.56; 2.03) | -1.10  (-5.43; 3.24) | -0.72  (-4.58; 3.13) | 0.10  (-3.85; 4.05) | 1.37  (-3.63; 6.37) | 0.79  (-4.18; 5.75) |
| Log peak HIV pVL at follow-up* | -0.51  (-2.15; 1.12) | -0.14  (-1.75; 1.48) | -0.16  (-1.62; 1.30) | 0.16  (-1.32; 1.64) | 0.17  (-1.75; 2.10) | 0.19  (-1.70; 2.07) |
| Use of cART before baseline (>3months: Yes vs. No) | **-4.69**  **(-9.29; -0.10)** | -2.91  (-8.27; 2.46) | -3.77  (-7.87; 0.32) | -2.16  (-7.06; 2.74) | -1.65  (-7.31; 4.01) | -0.95  (-7.45; 5.55) |
| Lifetime duration of cART use at baseline** (per 1 SD=142 weeks) | -0.33  (-2.86; 2.20) | 0.03  (-3.01; 3.07) | -0.00  (-0.02; 0.01) | -0.08  (-2.91; 2.75) | 0.83  (-2.62; 4.27) | -0.24  (-2.64; 2.17) |
| Duration of cART, baseline to follow-up  (per 1 SD=160 weeks)*** | -0.47  (-2.51; 1.56) | 0.59  (-1.77; 2.95) | -0.89  (-2.73; 0.95) | 0.08  (-2.12; 2.28) | -0.30  (-2.73; 2.14) | -0.13  (-3.09; 2.83) |
| Lifetime duration of cART use at follow-up** (per 1 SD=267 weeks) | -1.18  (-3.20; 0.84) | 0.01  (-2.75; 2.77) | -1.38  (-3.19; 0.43) | -0.33  (-2.88; 2.22) | -0.24  (-2.64; 2.17) | 0.13  (-3.18; 3.45) |
| TDF use, baseline to follow-up  (Yes vs. No) | -2.01  (-7.79; 3.76) | -2.04  (-7.69; 3.61) | -2.04  (-7.18; 3.10) | -2.11  (-7.29; 3.06) | -1.11  (-7.80; 5.59) | -4.53  (-10.97; 1.90) |
| Duration of TDF at follow-up  (per 1 SD=185 weeks) | 1.00  (-1.28; 3.28) | 0.95  (-1.65; 3.55) | -0.34  (-2.22; 1.55) | -0.16  (-2.25; 1.93) | -0.49  (-3.13; 2.16) | 0.20  (-2.79; 3.19) |
| Liver disease (Yes vs. No) | 2.83  (-1.28; 6.93) | 2.98  (-0.97; 6.93) | 1.49  (-2.19; 5.17) | 1.24  (-2.43; 4.91) | -0.69  (-5.49; 4.11) | -0.66  (-5.43; 4.10) |
| Menopausal status at follow-up  (menopausal vs. premenopausal) | **-5.19**  **(-9.05; -1.32)** | -3.87  (-10.24; 2.49) | **-4.58**  **(-8.01; -1.14)** | -5.57  (-11.25; 0.11) | **-5.58**  **(-10.08; -1.07)** | -2.51  (-10.60; 5.58) |
| BMI change (kg/m^2^) | 0.04  (-0.64; 0.72) | 0.13  (-0.53; 0.80) | **0.57**  **(0.03; 1.12)** | **0.64**  **(0.10; 1.17)** | -0.01  (-0.76; 0.75) | 0.00  (-0.73; 0.73) |

pVL – HIV plasma viral load; cART – combined antiretroviral therapy; TDF – tenofovir disoproxil fumarate; BMI – body mass index

*In WLWH with detectable HIV plasma viral load (pVL) or peak HIV pVL (i.e., > 40)

**In those using cART

***n=48

^Models were adjusted for baseline age, height and BMI.

**Supplemental Table 4**. **Number (%) of White women living with HIV (WLWH) and White HIV-negative controls who reported fractures at baseline and follow-up.** Bold text indicates a significant difference between White WLWH and controls.

| **Fracture type** | | **White WLWH**  **(n=29)** | **White Controls**  **(n=29)** | **Difference in proportion (95% CI)** |
| --- | --- | --- | --- | --- |
| **Prevalent (baseline)** | Any fragility fracture excluding hand, foot, skull | 9 (31.0%) | 3 (10.3%) | **20.7% (0.5; 40.9)** |
|  | Major osteoporotic fracture | 4 (13.8%) | 2 (6.9%) | 6.9% (-8.7; 22.5) |
| **Incident (follow-up period)** | Any fragility fracture excluding hand, foot, skull | 3 (10.7%) | 0 (0.0%) | 10.7% (-0.7; 22.2) |
|  | Major osteoporotic fracture | 3 (10.7%) | 0 (0.0%) | 10.7% (-0.7; 22.2) |

Major osteoporotic fracture = fragility fracture of the hip, forearm/wrist, clinical spine or humerus/shoulder fracture.
